# Association between a Primitive Brain Tumor and Cerebral Aspergillosis

**DOI:** 10.1155/2012/748202

**Published:** 2012-02-20

**Authors:** Siegfried Hélage, Charles Duyckaerts, Danielle Seilhean, Jean-Jacques Hauw, Jacques Chiras

**Affiliations:** ^1^Laboratoire de Neuropathologie Escourolle, Institut du Cerveau et de la Moelle épinière, Université Pierre et Marie Curie, Groupe Hospitalier Pitié-Salpêtrière, Assistance Publique-Hôpitaux de Paris, 47-83 boulevard de l'Hôpital, 75651 Paris cedex 13, France; ^2^Service de Neuroradiologie, Institut du Cerveau et de la Moelle épinière, Université Pierre et Marie Curie, Groupe Hospitalier Pitié-Salpêtrière, Assistance Publique-Hôpitaux de Paris, 47-83 boulevard de l'Hôpital, 75651 Paris cedex 13, France

## Abstract

Cerebral aspergillosis is a rare pathology of poor prognosis in spite of the use of adapted antifungal treatments. This infection of the central nervous system is generally the complication of an invasive aspergillosis with hematogenic scattering from pulmonary focal spots. It can arise in immunocompetent patients treated with prolonged corticotherapy or chemoradiotherapy for cancer. A case of lethal cerebral aspergillosis in a patient with an infiltrative glioma treated with corticotherapy and radiotherapy is reported. Clinicopathological aspects and therapeutic approach are described.

## 1. Introduction

Infections of the central nervous system (CNS) are major complications of antineoplastic treatments, mainly because of drugs that weaken the immune system, in particular corticotherapy and chemotherapy. During invasive aspergillosis, the cerebral location is a frequent complication. The prognosis is then unfavourable, with a mortality rate of about 86% [1–3]. It is mostly a postmortem diagnosis. The presentation of a cerebral aspergillosis is polymorphic: meningitis, meningoencephalitis, granuloma, brain abscess, and vasculitis. It can mimic cerebral tuberculosis, pyogenic abscess, or brain tumour. Symptoms are nonspecific; the patient can be apyretic [4–6]. Aspergillosis is an infection difficult to treat, especially in immunosuppressed patients. The fungus reaches the CNS by hematogenic scattering from peripheral focal spots; the portal of entry is mostly the lung [6]. A cerebral involvement due to an infected paranasal sinus is also possible. Occurrence of a cerebral aspergillosis in patients with an infiltrative glioma does not seem rare in view of our listed cases (Table 1). We choose to report here one of these clinicopathological histories (case A).

## 2. Case Presentation

A 66-year-old male, smoker, with diffuse atheromatosis, presented with intracranial hypertension, gradually increasing in gravity. A left occipital tumor was biopsied, leading to the discovery of a cerebral grade III-IV glioma (WHO 2007 classification) near the left ventricular atrium, not operable because of its critical location close to functional areas. Radiotherapy was decided to deliver 60 Gy in the tumor volume associated with corticotherapy. The treatment was well tolerated. Temozolomide chemotherapy was secondarily planned.

**Figure 1:**
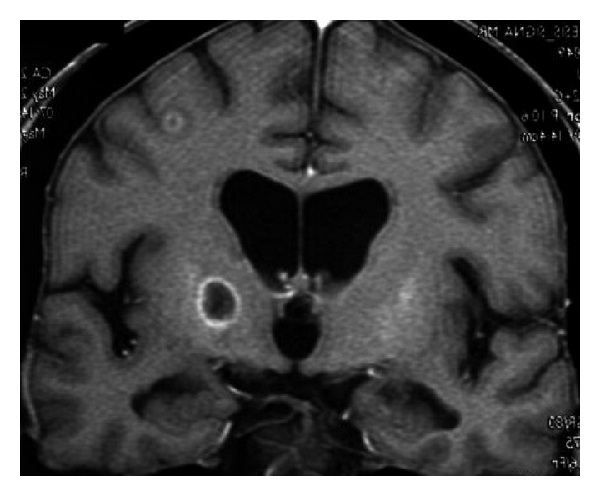
T1-weighted contrast-enhanced coronal MRI. Hypointense nodular lesion in the right capsulothalamic area, with peripheral annular enhancement. Small cortical-subcortical ipsilateral satellite lesion with annular enhancement and perilesional edema.

Mechanical left coxalgia and pygalgia appeared few weeks after the treatment. MRI was suggestive of aseptic osteonecrosis of the left femoral head of cortisonic origin. Increasing pain was concomitant with oedema of the left lower limb. Ultrasound revealed a left coxofemoral intra-articular effusion and eliminated venous thrombosis. A feverish syndrome appeared then, but bacteriological samples were negative. However, a neurological syndrome appeared, consisting of apathy, drowsiness, mnesic troubles, likely secondary to the cerebral radiotherapy and morphinic painkillers. The patient was transferred to the infectious disease unit, where a left hemiparesis was noticed. The brain CT scan showed multifocal hypodensities compatible with cerebral abscesses. The lumbar puncture did not show elevated cell count; the bacteriological examination was negative. A probabilistic biantibiotic therapy was established, in association with an intravenous antiherpetic treatment. Consciousness quickly degraded. A cerebral MRI was undertaken, finding images suggestive of multifocal abscesses (Figure 1).

The patient was transferred to the intensive care unit for septic shock. MRI of the left lower limb showed necrotizing fasciitis with myonecrosis. Multiple lung lesions suggestive of septic emboli were noticed on the thoracic CT scan. A surgical wound care was undertaken for an extensive cellulitis of the left lower limb.

During the infectious investigation, an aspergillar antigenaemia returned positive, confirmed on two other samples, suggestive of invasive aspergillosis. Mycological and parasitological examinations were negative for cryptococcosis and toxoplasmosis, in particular in the cerebrospinal fluid (CSF). The sample of necrotizing fasciitis was positive for polybacterial intestinal flora, compatible with anoperineal portal of entry. A polyantibiotic therapy was established, in association with the intravenous Voriconazole, in the hypothesis of aspergillar cerebral abscesses.

The course of septic shock was unfavourable; a syndrome of multivisceral failure developed. The follow-up cerebral CT scan found multiple round opacities with mass effect, suggestive of abscesses with midline deviation. The gravity of the situationbrought about canceling the cerebral microbiological biopsy. Death followed cardiocirculatory collapse.

In brief; this patient with cerebral high-grade glioma, under long-term corticosteroids, underwent a septic shock secondary to an extensive cellulitis of the left lower limb. A neurological syndrome with left hemiplegia and consciousness degradation was related to aspergillar cerebral abscesses with mass effect.

**Figure 2:**
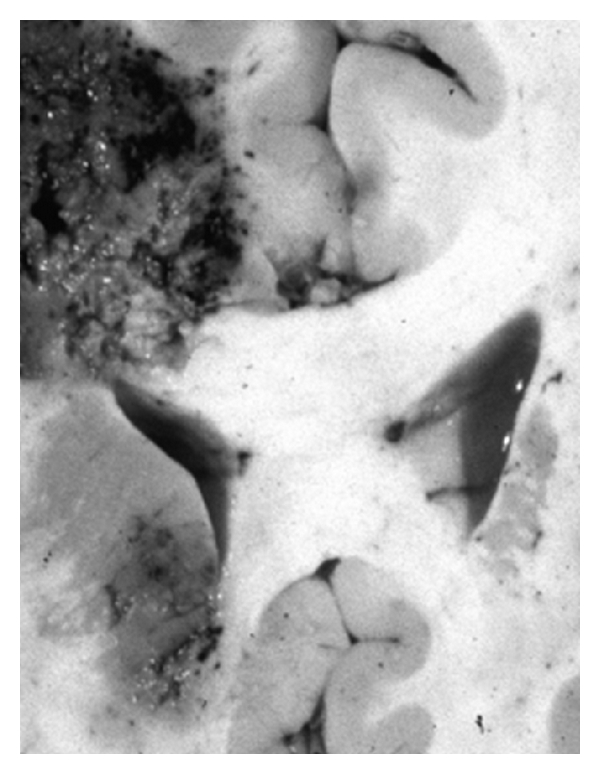
Autopsic brain coronal section. Right thalamus and corona radiata abscessed hemorrhagic infarcts.

**Figure 3:**
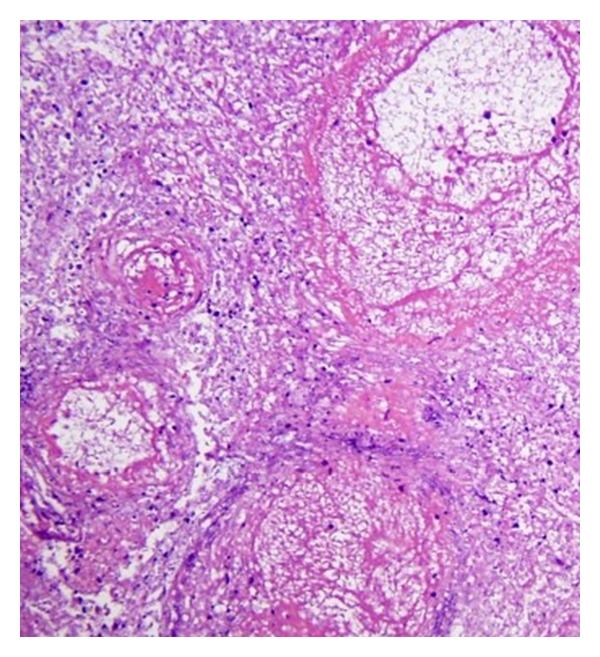
Corresponding histopathological section, stained with hematoxylin and eosin, showing hemorrhagic necrosis rich in altered polynuclear cells.

An autopsy was undertaken:

- on the infectious side, there were abscesses of both superior pulmonary lobes. The microscopic examination brought to light aspergillar abscesses with branched out Grocott-positive filaments, within necrotic tissue. An aspect of fasciitis of the left lower limb was also found. The macroscopic examination of the brain revealed an abscessed right frontal callosal mass (Figure 2) and satellite microabscesses separated from this main focal spot, associated with bilateral uncal herniation. During microscopy, we noticed zones of necrosis rich in altered polynuclear cells (Figure 3). PAS (periodic acid Schiff) and Grocott-positive mycelial strands were brought to light, often surrounded with polynuclear cells. Within these abscessed focal spots of mycelial origin, the branched-out aspect of filaments and their morphology suggested mycosis due to *Aspergillus fumigates*,
- on the carcinological side, we noticed a left hematic and necrotic parietooccipital lesion, compatible with an intratumoral hematoma. During microscopy, it appeared that the necrotic center was surrounded with an important glial reaction, within which neoangiogenesis and cells with large nuclei were observed. Macrophages loaded with hemosiderin were also observed,
- besides, a recent infarct was located in the lateral subdivision of the thalami, doubtless secondary to compression of the posterior cerebral arteries because of bilateral uncal herniation.

In brief; multiple pulmonary and cerebral aspergillar abscesses coexisted with a necroticohemorrhagic brain lesion, which was surrounded with neoangiogenesis, suggesting a tumor lesion reshaped by radiotherapy.

Note that this clinicopathological history matches with our other cases of listed gliomas, treated in a constant way by corticotherapy with an antiedematous aim (Table 1).

## 3. Discussion

Corticosteroids act on various stages of the immune response: they inhibit the presentation of antigens on the surface of monocytes/macrophages dependent on HLA class II histocompatibility antigens, the T-lymphocytes proliferation dependent on interleukin-1 (IL-1) and on IL-2, and the cytotoxicity (T and NK) that is dependent on interferon-gamma and on IL-2. Any prolonged cortisonic treatment of more than 15 days decreases immune functions, especially those carried out by T cells, while levels of antibodies synthesized by B-lymphocytes are little modified. Corticosteroids inhibit the migration of polynuclear neutrophils towards inflammatory sites and limit their apoptosis, explaining the polynucleosis classically observed under corticosteroid treatment [7]. We guess that, in most infectious diseases, a prolonged corticotherapy, by decreasing two fundamental means of defense, inflammation and immunity, can have catastrophic consequences. It is possible that the presence of a brain tumor is a cofactor favoring the development of cerebral aspergillosis, especially in the context of systemic immunosuppression. The possible local immunological disorders, associated with a greater permeability of the blood-brain barrier, could facilitate a fungal cerebral settlement in case of septicemia from a pulmonary focal spot.

*Aspergillus fumigatus* is the most common variety of *Aspergillus*. It is a commensal of the respiratory tract, which is the portal of entry. Cerebral aspergillosis is caused more frequently by *Aspergillus fumigatus*, in context of invasive aspergillosis with hematogenic scattering from pulmonary focal spots [6, 8]. In 90% of cases, the primary infectious site is the lung. The cerebral involvement affects 20% of patients with invasive aspergillosis [9]. Treatment with immunosuppressive drugs, prolonged corticotherapy, and radiochemotherapy in patients already weakened by an intercurrent pathology increases the probability of an aspergillar infection [2, 10, 11]. The diagnosis of cerebral aspergillosis is difficult because the inaugural symptoms are mostly not specific [3]: headaches, paralysis of cranial or somatic nerves, paresthesias, mental confusion, and/or epileptic seizures. Moreover, hemocultures and microbiological examination of the CSF are frequently negative [12]. Diagnostic certainty can be obtained by an aspiration biopsy of one of the abscesses after stereotactic location. Unfortunately, this gesture is not always practicable due to comorbidities such as severe underlying pathology or frequent pancytopenia and due to critical location of abscesses for example at the level of basal ganglia, with an important risk of severe complications. In this context we have indirect methods of diagnosis, the principle of which ensues from the very frequent scattering of aspergillosis in sites other than the CNS [13].

The presence of uni- or multifocal abscesses associated with vascular invasion leading to thrombosis is a characteristic feature of aspergillosis during neuropathologic examination. *Aspergillus* tends to invade arteries and veins because of its angiotropism, leading to necrotizing vasculitis, secondary thrombosis, and hemorrhage. There is often an infectious extension by contiguity [14]. The initially sterile infarcts can evolve into septic infarcts with formation of abscesses [15].

Aspergillosis generates typical wide septate filaments with dichotomous branching, associated with signs of vascular invasion, granulomatous formation, and giant cell reaction. Extension of fungal invasion in the neighbouring neuronal tissues and in blood vessels provokes hemorrhage, thrombosis, infarcts, necrosis, meningitis, and ventriculitis. This extension is at the origin of the varied clinicopathological aspects of cerebral aspergillosis. The neuropathological observations also depend on the depth of immunosuppression. In case of extreme immunosuppression like in bone marrow transplant or prolonged severe neutropenia, numerous aspergillar strands are found associated with badly bounded inflammation, constituted of some mononuclear and polynuclear cells. In case of less severe immunosuppression, inflammation is frank with frequent formation of granulomas constituted of lymphocytes, plasmocytes, and rare mycelial strands. Necrotic damage is frequent, whatever is the depth of immunodeficiency, confirming the vascular tropism of the pathogen.

Invasion of thalamoperforant and lenticulostriate arteries, responsible for thalamic and basal ganglia infarcts, suggests the diagnosis of cerebral aspergillosis, especially when the clinical context is evocative [14].

The most effective treatment of cerebral aspergillosis is medical and surgical. For a long period of time, the antifungal medication of reference was Amphotericin B, free or liposomal. Now, antifungal first-line treatment of invasive aspergillosis is Voriconazole, whose efficiency and tolerance are superior to Amphotericin B; its good intracerebral distribution justifies its first use in cerebral aspergillosis [16, 17]. The best results are obtained by associating antifungal medication with surgery of cerebral locations [18]. However, immunosuppression and deep critical location of these brain lesions make surgery rarely possible. The efficiency of intracavitary or intrathecal injection of Amphotericin B is not proved; it is exposed to severe iatrogenic complications such as meningitis, arachnoiditis, myelitis, or paralysis of cranial nerves [19].

Cerebral aspergillosis is of unfavourable prognosis. The mortality rate, which oscillates between 80 and 90%, is correlated with the time left before treatment starts. Aspergillosis abscesses in patients with progressive cancer are generally lethal [20]. Factors that favor the therapeutic efficacy are

- a unifocal and isolated character of the lesion, without scattering,
- absence of neurological signs,
- early diagnosis,
- preventive administration of an antifungal treatment in patients at risk for aspergillosis.

## 4. Conclusion

It would be advisable to keep in mind the risk of a fungal infection in any patient with a malignant tumor, including a cerebral tumor. Cerebral aspergillosis is a serious disease. This diagnosis should be suspected early to avoid a deleterious therapeutic delay, in particular in a context of immunosuppression, in the presence of pulmonary aspergillosis and typical location of lesions at the level of thalami and basal ganglia, particularly if there are multiple hemorrhagic infarcts on imaging.

## Figures and Tables

**Table 1:** List of cases diagnosed in our hospital between September 1997 and August 2008.

Case	Brain tumor type	Treatment type	Pulmonary aspergillosis occurrence	Cerebral aspergillosis occurrence
A	Grade III-IV glioma	Corticotherapy and radiotherapy	Yes	Yes
B	Glioblastoma	Corticotherapy, polychemotherapy, and radiotherapy	Yes	Yes
C	Grade III glioma (astrocytoma)	Corticotherapy	Yes	Yes
D	Grade III glioma (astrocytoma)	Corticotherapy	Yes	Yes
